# Comparative analysis of rodent lens morphometrics and biomechanical properties

**DOI:** 10.3389/fopht.2025.1562583

**Published:** 2025-04-04

**Authors:** Sepideh Cheheltani, Sadia T. Islam, Heather Malino, Kalekidan Abera, Sandeep Aryal, Karen Forbes, Justin Parreno, Velia M. Fowler

**Affiliations:** ^1^ Department of Biological Sciences, University of Delaware, Newark, DE, United States; ^2^ Department of Biomedical Engineering, University of Delaware, Newark, DE, United States

**Keywords:** lens stiffness, lens biomechanics, morphometrics, lens microstructures, allometry

## Abstract

**Introduction:**

Proper ocular lens function requires biomechanical flexibility, which is reduced during aging. As increasing lens size has been shown to correlate with lens biomechanical stiffness in aging, we tested the hypothesis that whole lens size determines gross biomechanical stiffness by comparing lenses of varying sizes from three rodent species (mice, rats, and guinea pigs).

**Methods:**

Coverslip compression assay was performed to measure whole lens biomechanics. Whole mount staining on fixed lenses, followed by confocal microscopy, was conducted to measure lens microstructures.

**Results:**

Among the three species, guinea pig lenses are the largest, rat lenses are smaller than guinea pig lenses, and mouse lenses are the smallest of the three. We found that rat and guinea pig lenses are stiffer than the much smaller mouse lenses. However, despite guinea pig lenses being larger than rat lenses, whole lens stiffness between guinea pigs and rats is not different. This refutes our hypothesis and indicates that lens size does not solely determine lens stiffness. We next compared lens microstructures, including nuclear size, capsule thickness, epithelial cell area, fiber cell widths, and suture organization between mice, rats, and guinea pigs. The lens nucleus is the largest in guinea pigs, followed by rats, and mice. However, the rat nucleus occupies a larger fraction of the lens. Both lens capsule thickness and fiber cell widths are the largest in guinea pigs, followed by mice and then rats. Epithelial cells are the largest in guinea pigs, and there are no differences between mice and rats. In addition, the lens suture shape appears similar across all three species.

**Discussion:**

Overall, our data indicates that whole lens size and microstructure morphometrics do not correlate with lens stiffness, indicating that factors contributing to lens biomechanics are complex and likely multifactorial.

## 1 Introduction

The ocular lens is a clear transparent tissue that is responsible for fine-focusing light onto the retina. The lens is composed of epithelial and fiber cells and is surrounded by a basement membrane called the capsule (1–3). The lens is a unique tissue in that it grows continually throughout life (4, 5). This occurs through lifelong proliferation of epithelial cells, and their differentiation into new fiber cells. These newly formed fiber cells add onto existing generations of fiber cells, resulting in concentric layers of fiber cells forming a radial gradient corresponding to cell age (6–8). Newly formed fiber cells reside at the cortical region of the lens, while the oldest fiber cells that formed the lens during embryonic development, are at the core of the lens. Studies show that this continued accumulation of fiber cells results in increasing lens volume and weight with animal age (9–12).

Previous allometric studies show a wide distribution of lens sizes across more than one hundred of animal species (9, 10). In general, most species exhibited negative allometry, where larger animals have proportionally smaller lenses (9). Like human lenses, those from many different animal species also show continuous growth in size with age (4, 10, 12). However, the lens does not increase in size indefinitely as the growth rate seems to plateau in older animals, likely due to space and size limitations within the eye (10, 12). We have previously performed an in-depth examination of lenses of wildtype mice of various ages that showed that mouse lens volume increases and becomes slightly more spherical with aging (13). Similar to lenses from humans, primates, and other rodents, mouse lenses also become stiffer with aging (13–22). Whether increasing lens size and changes in microstructural components with aging contribute to lens stiffness is not known.

As both lens size and biomechanical stiffness seem to scale with aging, it is conceivable that lens size may contribute to lens mechanical properties (4, 10, 12–22). However, lens size may not solely account for whole lens stiffness as previous studies have shown other lens structures can determine whole lens mechanical properties. For example, removal of the porcine lens capsule significantly decreases the whole lens stiffness and shear viscosity, indicating that the lens capsule plays a crucial role in determining whole lens biomechanical properties (23). Age-dependent changes in microstructural and cellular features, such as lens capsule thickening, epithelial cell area expansion, fiber cell widening, and loss of Y-shape suture, suggest potential contributions to lens stiffening with growth during aging (13, 24). It has also been suggested that the central core of the lens (nucleus) is an important determinant of lens biomechanical properties and ability for shape change (13, 14, 25). The lens nucleus is formed via compaction of fiber cells, resulting in a hardened lens core that is orders of magnitude greater in stiffness than the cortical fiber cells (14). Since the lens nucleus becomes larger and stiffer with aging in humans and mice, it is proposed to be responsible for whole lens stiffening with size and age (13, 14).

In this study, we systematically compared lens stiffness and microstructural features across three species with variations in lens size: mice, rats and guinea pigs. We selected mice, rats, and guinea pigs as representative rodent models with distinct differences in lens size and body mass, enabling an investigation of whether lens biomechanical properties scale with size. Additionally, these species are commonly used in lens research, making comparisons between them particularly relevant. In this study, we tested the hypothesis that lens size is a determinant of lens stiffness and investigated which structural features in the lens might be correlated with size and stiffness. To test this hypothesis, we used an allometric approach by systematically comparing the morphometric and biomechanical properties of lenses from three rodent species – mouse, rat, and guinea pig. Since lens size scales proportionally with organism size, this approach enabled us to investigate the relationship between lens size and biomechanical properties. Our study reveals that the biomechanical properties of lenses from different species are determined by complex multifactorial properties that are not related directly to either lens size or microstructure dimensions.

## 2 Materials and methods

### 2.1 Rodent lens dissection

Long Evans Rat and Guinea pig eyes from animals between the ages of 7 and 10 weeks were purchased (BioChemed Services; Winchester, VA). Eyes were shipped overnight in Phosphate-Buffered Saline (PBS, Sigma-Aldrich, P3813, pH 7.4) in conical tubes. The eyes were kept cold by surrounding the conical tubes with cooling packs in a sealed Styrofoam container.

Mouse care and euthanasia procedures were approved by the Institutional Animal Care and Use Committee at the University of Delaware. All procedures were conducted in accordance with the Association for Research in Vision and Ophthalmic and Vision Research (ARVO) Statement for the use of Ophthalmic and Vision Research, and the Guide for the Care and Use of Laboratory Animals by the National Institutes of Health. Mouse eyes were enucleated from wild-type C57BL/6 mice between the ages of 7 and 10 weeks. The mouse eyes were kept cold by surrounding the conical tubes with cooling packs in a sealed Styrofoam container to resemble the shipping conditions of the rat and guinea pig lenses.

Lenses were dissected from eyeballs in PBS (Quality Biologicals, Cat# 114-058-101, pH 7.4) as previously described (26). Briefly, the optic nerve was removed from the eyeballs using microdissection scissors, which were then used to cut from the posterior to the anterior region of the eyes. Finally, the lenses were released by applying pressure to the uncut sides of the eye.

### 2.2 Biomechanical testing of lenses and imaging

The compressive properties of lenses were assessed using load-controlled, sequential application of glass coverslips onto lenses as previously performed (13, 26–28) with minor modifications. Briefly, dissected lenses were placed into the 200 μm (for mice) or 300 μm (for rats and guinea pigs) deep divot within a bespoke loading chamber to accommodate the lens size of each species (27). Coverslip loads, with an average weight of 121.73 mg, were applied sequentially, two coverslips at a time, onto the lenses. In previous studies, a single coverslip was placed each time onto the lens (13, 26, 28). In this study, two coverslips per step was implemented to standardize compression measurements across species with larger lenses, such as those of guinea pigs, enabling more direct comparisons.

To allow for stress-relaxation equilibration, the lenses were compressed for 2 minutes prior to each image acquisition. Lenses were imaged at three different stages: 1. Before applying any load, 2. During compression after a 2-minute equilibrium period, 3. After load removal. Side view images were captured using a 45° angled mirror that was placed at a fixed distance from the lens, with a Swiftcam 20 Megapixel camera connected to an Olympus SZ11 dissecting microscope. After the removal of the final 20 coverslip load (2,434.60 mg), the wet weights of lenses were measured using an analytical weighing scale (Mettler-Toledo; Columbus, OH). Finally, the hardened nuclear masses (center core region) of the lens were isolated. This was achieved by removing the lens capsule and dissociating the soft cortical fiber cells by gently rubbing between gloved fingertips. The remaining tissue was considered to be lens nucleus. Digital images were then obtained as described above.

### 2.3 Gross lens morphometric analysis of images

The axial and equatorial diameters of lenses and nuclear regions were measured using FIJI software. Lens strain was calculated using the equation ϵ = (d-d_0_)/d_0_, where ϵ is strain, d is axial or equatorial diameter at a given load, and d_0_ is the initial axial or equatorial diameter before the application of any load (0 coverslips). Lens and nucleus volume were calculated using the equation, volume = 4/3 x π x r_E_^2^ x r_A_, where r_E_ is the equatorial radius and r_A_ is the axial radius. Lens aspect ratio was a ratio of the equatorial to axial diameter. Nuclear fraction was calculated using the formula, nuclear fraction = nuclear volume/lens volume.

### 2.4 Whole mount imaging preparation of fixed lenses

Whole mount imaging was performed as previously described (26). Dissected lenses were fixed in 4% formaldehyde in PBS at room temperature. After 30 minutes, lenses were washed three times (5 minutes per wash) in PBS and then incubated in permeabilization/blocking solution (PBS containing 0.3% Triton, 3% bovine serum albumin, and 3% goat serum) at room temperature for another 30 minutes. Next, lenses were placed in staining solution containing fluorescent CF640 dye conjugated to wheat-germ agglutinin (Biotium, Fremont, CA) (1:500), Hoechst 33342 (Biotium) (1:500), and rhodamine-phalloidin (Thermo Fisher Scientific) (1:20). After an overnight incubation at 4°C, lenses were washed three times in PBS for 5 minutes before performing confocal microscopy. The staining was performed in 1.7 mL Eppendorf tubes, and lenses were handled/transferred using forceps designed for mouse embryos (Hammacher instrumente, #HSC 702-93, Germany) to prevent damage.

### 2.5 Confocal microscopy

Confocal microscopy on whole lenses was performed on a Zeiss LSM880 laser-scanning confocal fluorescence inverted microscope (Zeiss, Germany) as previously described (26). To image the anterior capsule and epithelial cells, lenses were placed anterior side down on 10 mm microwell glass-bottomed dishes (MakTek, Ashland, MA). To prevent lens movement while imaging, the lenses were immobilized within a small circular divot that was created in a thin layer of 2% agarose in PBS using a 2mm biopsy punch (Accu-punch, Acuderm inc., Fort Lauderdale, FL). To image the anterior and posterior sutures, lenses were placed with the anterior or posterior side down within the agarose divot. Z-stack images were acquired with a 40x oil Plan-Apo 1.4 NA objective using a step size of 0.3μm. To image the equatorial fiber cells, the lenses were placed in optical glass-bottomed Fluorodishes (World Precision Instruments, Sarasota, FL) and balanced on the side using agarose wedges (26, 29). Z-stack images were acquired with a 20x air 0.8 NA objective using a step size of 0.5μm.

### 2.6 Analysis of microstructural and cellular features

Raw fluorescent images were processed using Zen Black 2.3SP1 (Zeiss) software. FIJI software was used for lens morphometric analysis and measurements of microstructural features as previously described (26). Anterior capsule thickness was measured by obtaining intensity distributions of capsular (WGA-640) and basal epithelial F-actin (rhodamine-phalloidin) stains using line scan analysis of XZ plane-view reconstructions of the lens anterior and by performing subtractive peak-to-peak analysis of fluorescent pixel intensity to obtain distance (26, 30).

Epithelial cell area was calculated by tracing a population of at least 30 cells (region of interest; ROI) whose boundaries were identified by staining of F-actin, using rhodamine-phalloidin, at cell membranes. The total number of cells within the ROI was determined by counting cell nuclei stained with Hoechst. Average cell number was calculated using the equation, average cell area = ROI area/total number of cells (30).

Fiber cell width was calculated by line scan analysis of fiber cell membranes stained with rhodamine-phalloidin at the lens equator, ~10μm inward from the fulcrum (26, 30). On FIJI, the Distributed Deconvolution (Ddecon) plugin with Z-line predictive model was used to provide high spatial precision when analyzing fiber cell widths.

### 2.7 Statistical analysis

Each experiment was conducted using at least four biological replicates. (4-19 lenses depending on the experiment). The sample size (N) of each experiment is indicated in the figure legend. Differences between multiple groups of data (lens mechanical stiffness) were assessed using a two-way repeated measures analysis of variance followed by Tukey’s multiple comparisons *post hoc* test. Differences between the three groups of data were detected using one-way ANOVA. All analysis was performed and graphs were made using GraphPad Prism and Microsoft Excel.

## 3 Results

### 3.1 Lens size scales with rodent size

**Figure 1:**
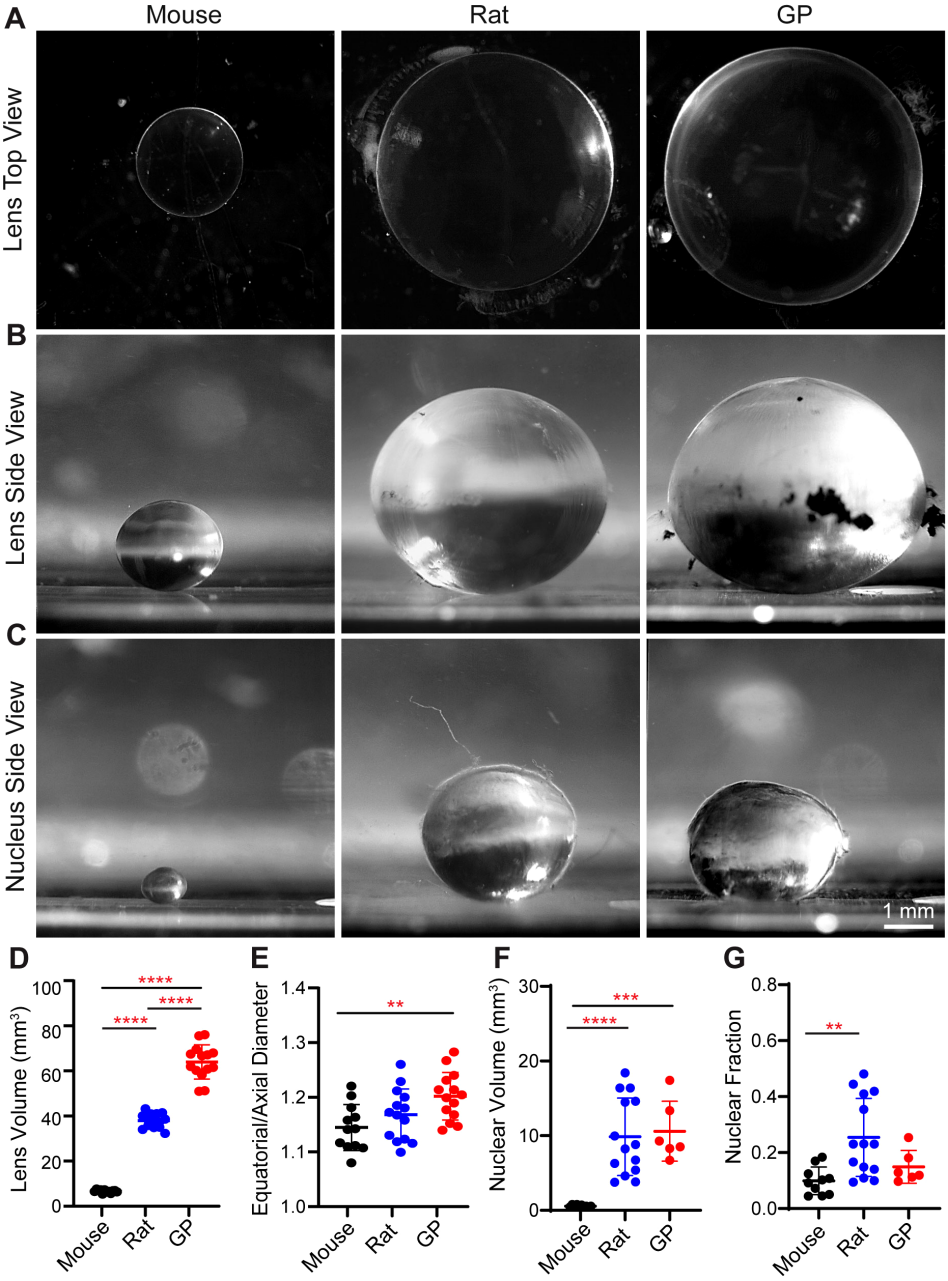
Lens and nucleus size are significantly different among different rodent models. **(A, B)** Top view and side view images of lenses and **(C)** side view images of lens nucleus from mouse, rat, and guinea pig (GP). Scale, 1 mm. **(D)** Calculated gross lens volumes, **(E)** equatorial to axial diameter ratio of lenses (aspect ratio), **(F)** Calculated nuclear volume and **(G)** nucleus to lens fraction in mouse, rat, and GP. N= 6-14 lenses per species. **p <0.01: ***p <0.001 ****p < 0.0001.

To characterize the allometric properties of rodent lenses, we used 7-10 weeks old mice, rats, and guinea pigs. The mice are the smallest of the rodents, with an average weight of 28.1 ± 0.6 g. Rats have an average weight of 252.2 ± 25.0 g, which is 9.0-fold more than the mice. Guinea pigs weigh the most of the three species, averaging 571.3 ± 33.9 g, which is 20.3-fold more than the mice and 2.3-fold more than the rats. Next, we dissected the lenses (**Figure 1A**) and measured their wet weight (**Table 1**). Mouse lenses weigh the least, with an average wet weight of 5.8 ± 0.01 mg. Rat lenses had a significantly higher wet weight, averaging 43.3 ± 15.69 mg, approximately 7.5 times heavier than mouse lenses. Guinea pig lenses were the heaviest, with an average wet weight of 74.7 ± 4.90 mg, which is 12.8-fold heavier than mouse lenses and 1.7-fold heavier than rat lenses. Overall, the wet lens weights from mice, rats, and guinea pigs account for 0.020%, 0.017%, and 0.013% of total body weights, respectively.

**Table 1:** Summary of mouse, rat and guinea pig lens morphology measurements.

Morphometrics	Mouse	Rat	Guinea Pig
**Animal weight**	28.1 + 0.6 g (4)	252.2 + 25.0 g (6)	571.3 + 33.9 g (6)
**Lens wet weight**	5.80 + 0.13 mg (8)	43.29 + 15.69 mg (11)	74.68 + 4.90 mg (10)
**Lens axial diameter**	2.08 + 0.05 mm (12)	3.76 + 0.15 mm (14)	4.39 + 0.24 mm (14)
**Lens equatorial diameter**	2.35 + 0.09 mm (12)	4.39 + 0.13 mm (14)	5.27 + 0.19 mm (14)
**Lens volume**	6.66 + 0.69 mm (12)	37.94 + 3.19 mm (14)	63.95 + 7.61 mm (14)
**Lens aspect ratio**	1.15 + 0.04 (12)	1.17 + 0.05 (14)	1.20 + 0.04 (14)
**Nuclear volume**	0.56 + 0.18 mm^3^ (10)	9.85 + 5.19 mm^3^ (14)	10.59 + 4.01 mm^3^ (6)
**Nuclear fraction**	0.10 + 0.05 (10)	0.25 + 0.14 (14)	0.15 + 0.06 (6)
**Capsule thickness**	11.10 + 0.71 μm (6)	8.07 + 1.72 μm (6)	14.80 + 2.14 μm (6)
**Anterior epithelial cell area**	144.7 + 14.7 μm^2^ (4)	180.3 + 34.9 μm^2^ (5)	346.7 + 28.2 μm^2^ (6)
**Fiber cell width**	11.30 + 0.47 μm (7)	8.70 + 0.44 μm (4)	13.79 + 0.91 μm (7)

All data are represented as mean ± standard deviation. The sample size is indicated in parentheses.

Next, we measured the axial and equatorial diameter of lenses and lens nucleus using side view images (**Figures 1B, C**) to calculate volume. Mouse lenses are the smallest of the three species, with an average volume of 6.66 ± 0.69 mm^3^ (**Figure 1D**; **Table 1**). Rat lenses have an average volume of 37.94 ± 3.19 mm^3,^ which is ~5.7-fold greater than mouse lenses. Guinea pig lenses are the largest of the three species with an average volume of 63.95 ± 7.61 mm^3^, which is ~9.6-fold larger than mouse lenses and ~1.7-fold larger than rat lenses (**Figure 1D**; **Table 1**). In addition, we calculated the lens aspect ratio, revealing that guinea pig lenses have a significantly higher equatorial to axial diameter ratio than mouse lenses (**Figure 1E**; **Table 1**).

Next, we investigated potential differences in nuclear size between mouse, rat, and guinea pig lenses by isolating the lens nuclei and calculating their volumes by measuring axial and equatorial diameters. Our findings revealed that the nuclear volumes of rat and guinea pig lenses are significantly larger than those of mouse lenses (**Figures 1C, F**; **Table 1**). However, the nuclear volume to whole lens volume ratio (nuclear fraction) is only significantly different between mouse and rat lenses and still is not different between rat and guinea pig or between mouse and guinea pig (**Figure 1G**).

### 3.2 Lens biomechanical stiffness is independent of lens size

As the rat and guinea pig lenses were shipped overnight in PBS while mouse lenses were freshly dissected for this study, we first determined whether storage affected lens biomechanical properties. We compared the biomechanical properties of mouse lenses that were dissected from whole eyes immediately after animal sacrifice or that were dissected from whole eyes that had been stored overnight in cold PBS. Our analysis revealed no significant differences in mechanical properties between lenses that were freshly dissected or lenses dissected from stored eyes (**Supplementary Figure 1**).

**Figure 2:**
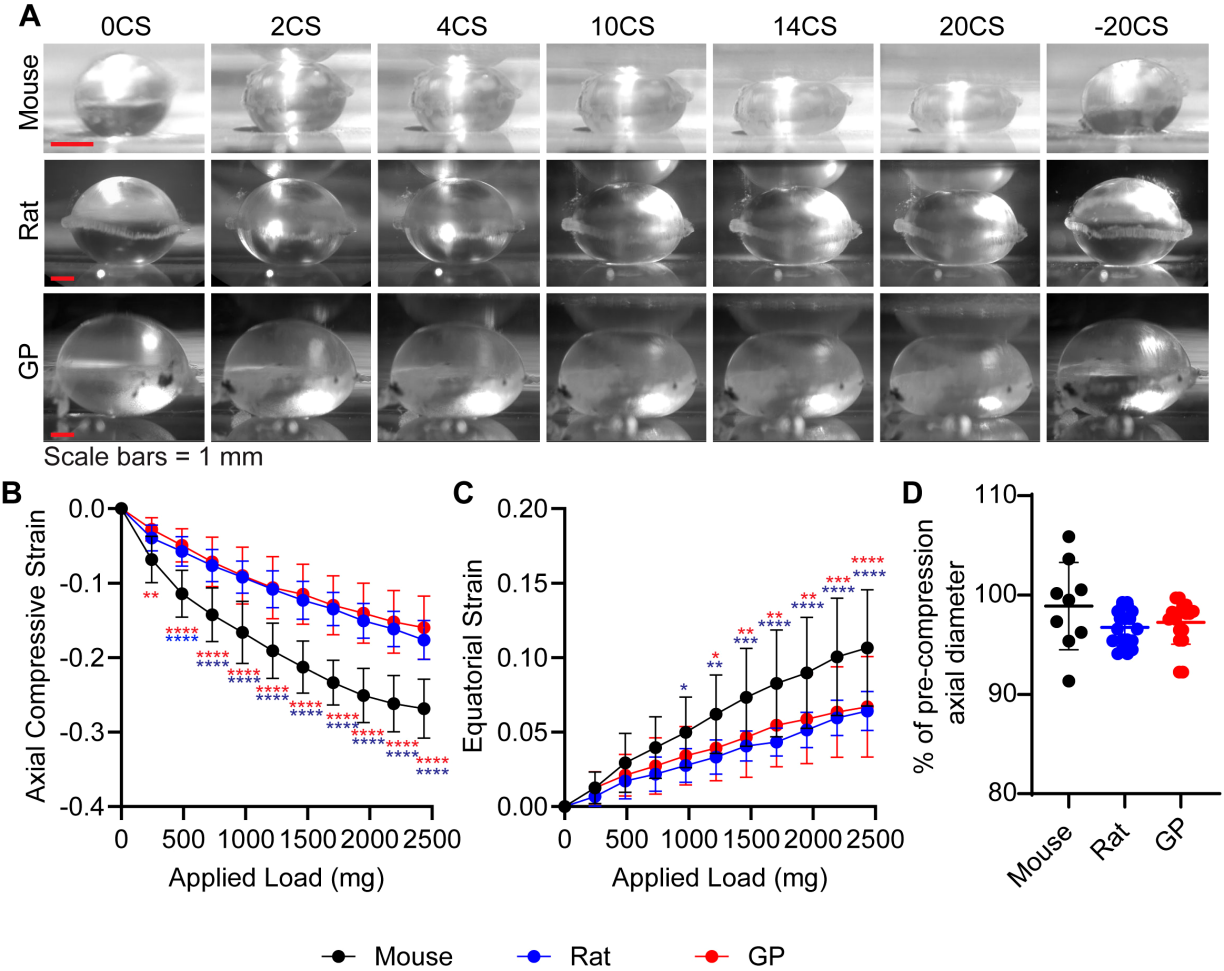
Mouse lenses are significantly softer than rat and guinea pig lenses. **(A)** Side-view images of mouse, rat, and guinea pig (GP) lenses captured during the coverslip compression assay, under varying levels of compression: 0, 2, 4, 10, 14, 20, and the release of 20 coverslips (-20 CS). Scale bars, 1 mm. The calculated **(B)** axial and **(C)** equatorial strain of the lenses under compression indicate that rat and GP lenses exhibit similar biomechanical properties, whereas mouse lenses are notably softer in comparison to both. N= 10-13 lenses per species. **(D)** % of pre-compression axial diameter reveal that the rodent lenses can recover to near their original size after the removal of 20 coverslips. N=9-19 lenses per species. *p < 0.05; **p < 0.01; ***p < 0.001; ****p < 0.0001. Blue and red asterisks indicate that mouse lenses are significantly different from rat and GP lenses, respectively.

To examine if lens biomechanical stiffness scales with lens size, we performed load-controlled (coverslip) compression of lenses followed by calculation of axial compressive strain (negative) and equatorial expansion strain (positive) (**Figure 2**). Based on their larger size, we hypothesized guinea pig lenses would be the stiffest of the three species, followed by rat then mouse. At both low and high loads, we observe a significantly greater axial compression in mouse lenses compared to both rat and guinea pig lenses (**Figure 2B**). While the equatorial strain is not different at lower loads, mouse lenses show a significantly greater equatorial expansion at higher loads compared to rat and guinea pig lenses, indicating greater equatorial strain (**Figure 2C**). Calculated axial and equatorial strains indicate that mouse lenses are the softest, while no difference was observed between rat and guinea pig lens stiffness. Interestingly, when we removed the 20 coverslips, lenses from all three rodents were able to recover to their original shape (**Figure 2D**). Our data indicate that lens biomechanical stiffness does not necessarily scale with lens size.

Previously, we found that the dimensions of certain lens microstructural and cellular features, such as capsule thickness, epithelial cell area, fiber widths, and nucleus size, increase as the lens continues to grow in size with age, along with alterations in suture morphology (13). These age-related changes in lens microstructural features prompted us to question whether differences in these microstructural/cellular features could account for differences in lens stiffness between species. Therefore, we compared the microstructural and cellular features of lenses from mice, rats, and guinea pigs

### 3.3 Assessment of lens microstructural features in mice, rats, and guinea pigs

**Figure 3:**
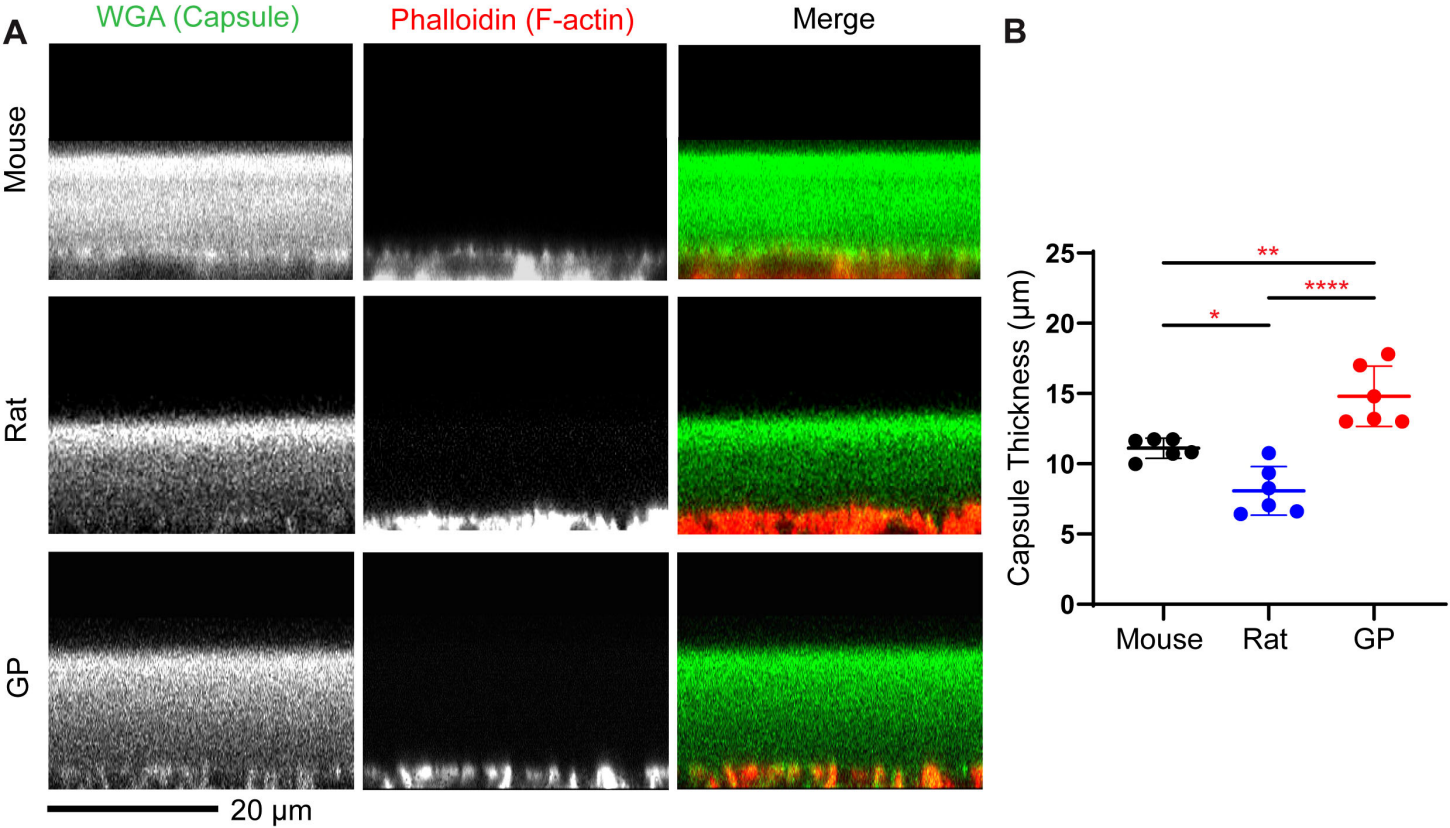
Capsule thickness is significantly different among different rodent models. Fixed lensed were labeled with WGA (green) for lens capsule and phalloidin (red) to visualize basal F-actin in epithelial cells at the epithelial-capsule interface. **(A)** representative (XZ reconstruction) confocal images of the mouse, rat, and guinea pig (GP) anterior lens capsule. Scale bar, 20 µm. **(B)** The anterior capsule is significantly thicker in GP compared to mice and rats, with rats having the thinnest anterior capsule among the three species. The plot represents the mean ± SD of 6 lenses per species. *p<0.05; **p<0.01; ****p < 0.0001.

We analyzed lens capsule and cell sizes in mouse, rat, and guinea pig lenses. Whole lenses were fixed and incubated in WGA to stain the lens capsule matrix (26), and Phalloidin to stain the F-actin associated with the epithelial cell basal membranes (**Figure 3A**). This allows us to measure the anterior capsule thickness by performing line scan analysis on sagittal (XZ plane) optical sections of reconstructed images (13, 26). We determined that mouse lenses have an average capsule thickness of 11.10 + 0.7 μm, while guinea pigs have an average lens capsule thickness of 14.8 ± 2.1 μm. Surprisingly, rat lenses have the thinnest capsule with an average capsule thickness of 8.1 ± 1.7 μm (**Figure 3B** and **Table 1**)

**Figure 4:**
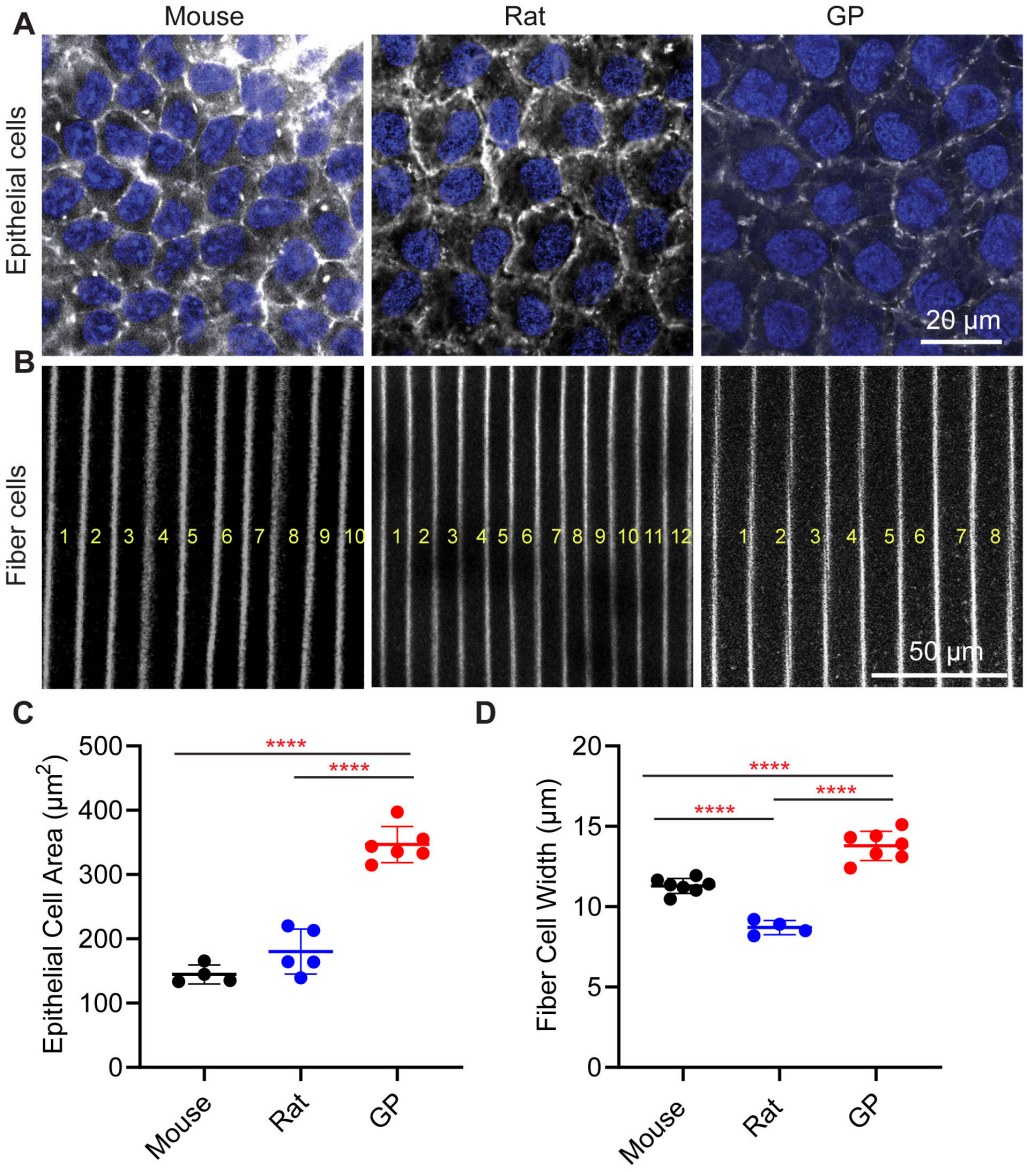
Lens epithelial cell and fiber cell widths are greater in guinea pig lenses. Fixed whole lenses were labeled with phalloidin (gray scale) to visualize F-actin at cell boundaries, and Hoechst (blue) to visualize epithelial cell nuclei. Representative confocal images of **(A)** anterior epithelial cells and **(B)** equatorial fiber cells from mouse, rat, and guinea pig (GP) lenses. In B, the yellow letter indicates the number of fiber cells. **(C)** The average anterior epithelial cell area measurements reveal that GP cell area is significantly higher than both mouse and rat cell area. **(D)** Fiber cell width measurements reveal that GP lenses have wider fiber cells compared to mouse and rat lenses. Additionally, mouse fiber cell widths are significantly greater than those of rat lenses. N=4-6 lenses per species. ****p < 0.0001.

Underlying the capsule at the anterior region of the lens are the epithelial cells. Phalloidin staining also allows us to visualize F-actin associated with cell membranes at the lateral boundaries of the anterior epithelial cells (**Figure 4A**), enabling us to measure epithelial cell areas. We found that the average epithelial cell area was 144.7 + 14.7 μm^2^ for mouse lenses, 180.3 + 34.9 μm^2^ for rat lenses, and 346.7 ± 28.2 μm^2^ for guinea pig lenses (**Figures 4A, C**). Thus, while anterior epithelial cell sizes did not differ significantly between mice and rats, they were approximately 2.4 times larger in guinea pig lenses compared to mouse lenses and 1.9 times larger in guinea pig compared to rat lenses. Therefore, the anterior epithelial cell area appears to scale with relative lens size in mice, rats, and guinea pigs, similar to previous observations of epithelial cell size scaling with increased lens size during mouse lens aging (13, 24).

Next, we measured the cortical fiber cell widths at the equator in three rodent lenses (**Figures 4B, D**). The fiber cell widths appear smallest in the rats, followed by mice, and then guinea pigs as there are more fiber cells in rat lenses within the same region of interest compared to mice and guinea pigs (**Figure 4B**). We observed that cortical fiber cells in mouse lenses have an average width of 11.30 + 0.47 μm, whereas those in guinea pigs have an average fiber cell width of 13.79 + 0.91 μm. The smallest fiber cell width is observed in rat lenses, with an average of 8.70 + 0.44 μm (**Figure 4D**; **Table 1**). Thus, while the epithelial cell areas scale with the lens size for mice, rats, and guinea pigs, the lens capsule thickness and fiber cell widths do not scale with size (**Figures 3**, **4**).

**Figure 5:**
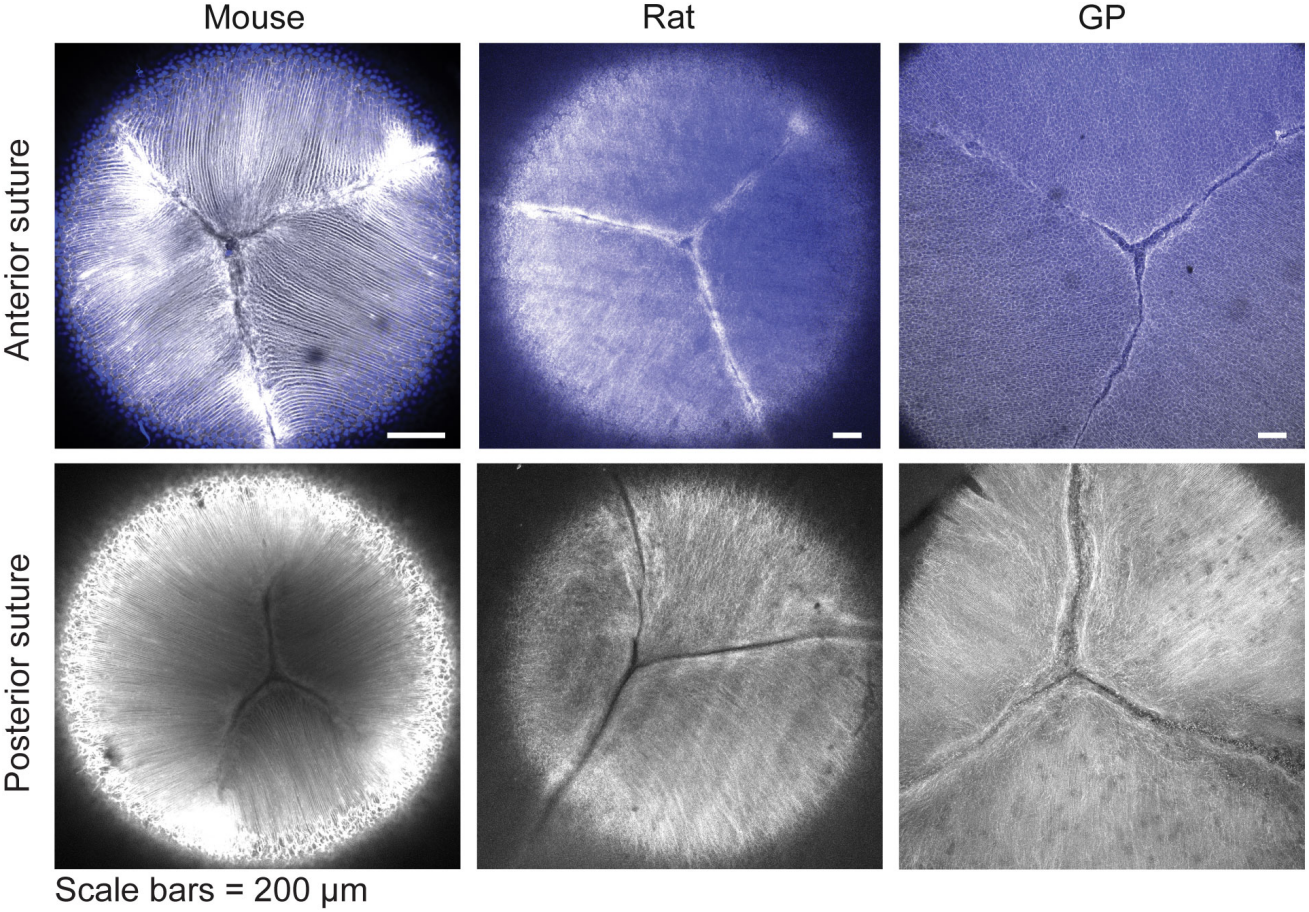
Suture organization in mouse rat and guinea pig lenses are similar. Lenses were stained with phalloidin (grayscale) to stain F-actin at cell boundaries and Hoechst (blue) to stain nuclei (only present at the anterior region of the lens). This revealed that Y-shaped sutures at the lens’s anterior (top panels) and posterior (bottom panels) regions are similar in mouse, rat, and guinea pig (GP) lenses. Scale bars, 100 µm.

Finally, since a high level of suture branching, as seen in primate lenses, may be indicative of lens flexibility and propensity for lens shape change (7, 31–33), we examined whether suture branching was different between mouse, rat, and guinea pig lenses. Our analysis revealed that the anterior and posterior suture organization in these species is similar, with 3-4 suture branches present in both regions of the lens (**Figure 5**).

## 4 Discussion

**Figure 6:**
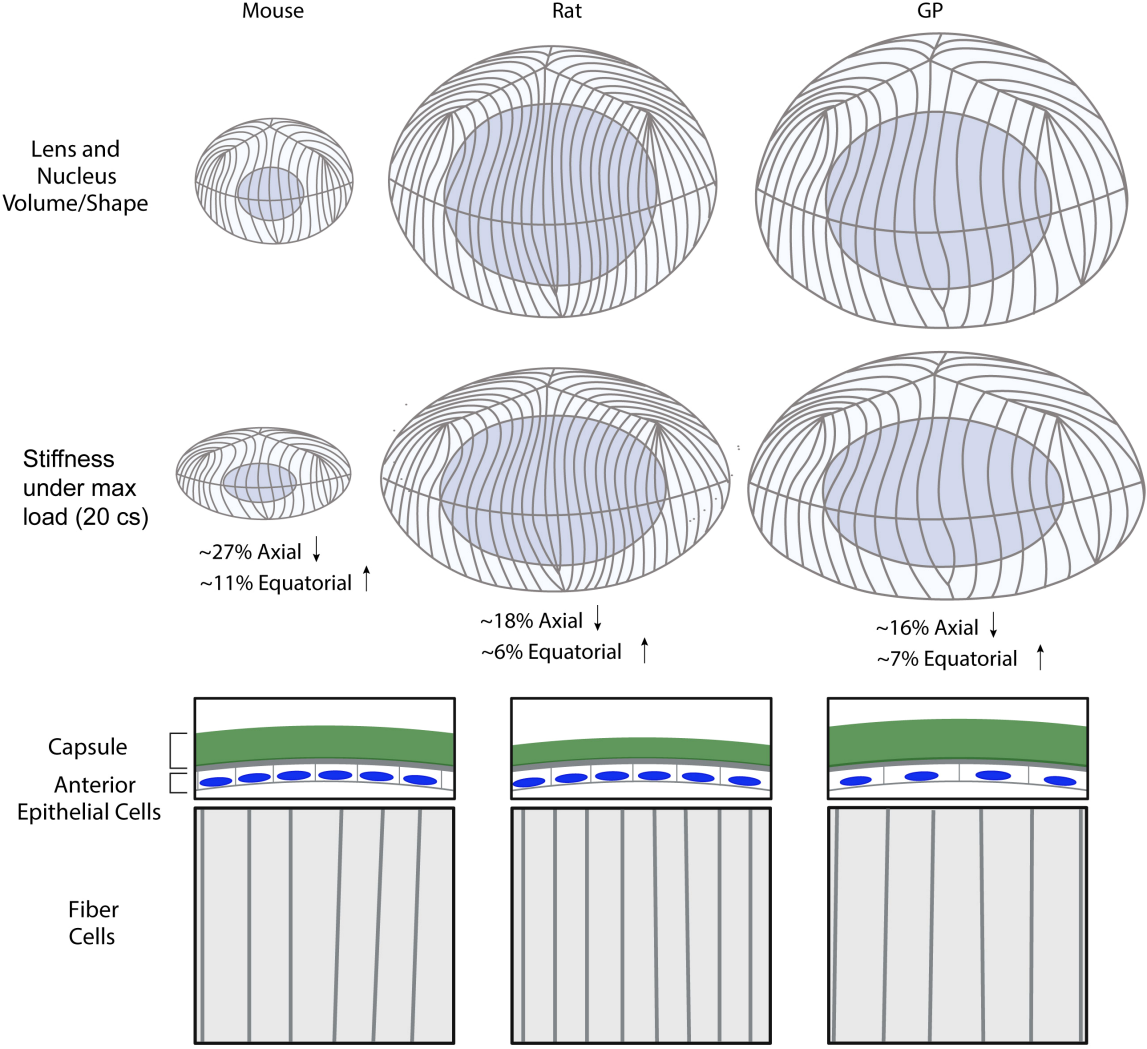
Summary diagram of the lens morphometrics and microstructural features in mouse, rat, and guinea pig. Lens volume is smallest in the mouse, followed by the rat and guinea pig (GP), while the nuclear fraction is highest in the rat. The mouse lens exhibits the highest axial and equatorial strain under compressive load, indicating a softer lens compared to the rat and GP. The lens capsule is thickest in the GP and thinnest in the rat. Anterior epithelial cell area is largest in the GP, while fiber cell width is smallest in the rat, followed by the mouse and then the GP. Created with BioRender.com.

In this study, we tested the hypothesis that lens biomechanical stiffness scaled with lens size and/or microstructures by comparing the whole lens morphology, biomechanics, and microstructural features of 7-10 weeks old mice, rats, and guinea pigs. Our findings are summarized in **Figure 6**, which reveals that mouse lenses, which are considerably smaller than those of rats and guinea pigs, are also mechanically softer (**Figures 1**, **2**, **6**). However, while guinea pig lenses are significantly larger than rat lenses (**Figures 1**, **2**, **6**), there is no difference in whole lens biomechanical properties between rat and guinea pig lenses, suggesting that lens size does not account for lens biomechanical properties across different rodent species. This raises the question- what lens features contribute to a stiffer lens in rats and guinea pigs compared to mice?

Previously, we have shown that mouse lenses become larger and stiffer with aging (13). Age-related changes in lens microstructure components, such as lens capsule thickening, epithelial cell area expansion, fiber cell widening, and enlargement of the lens nucleus, are all correlated with increased lens stiffness in mice (13, 34, 35). While previous research has shown that epithelial cell area increases with lens size and age in mice (13, 24), our findings indicate that this feature does not scale with lens size across species. Specifically, although mice had the smallest epithelial cell area and guinea pigs had the largest, there was no difference in epithelial cell area between mice and rats despite the increased size of rat lenses compared to mouse lenses (**Figure 4**).

The increase in fiber cell width and capsule thickness is hypothesized to contribute to an increase in lens stiffness with aging (13). However, our results showed no correlation between these parameters and lens size or stiffness (**Figures 3**, **4**). Rats had the thinnest capsule and narrowest fiber cells, while guinea pigs had the thickest capsule and widest fiber cells, yet both rat and guinea pig lenses had similar biomechanical stiffness (**Figures 3**, **4**). It is worth noting that mice from different genetic backgrounds have different capsule thicknesses (35). For example, 2 months old mice of 129X1 background have an average capsule thickness of 7.0 ± 0.6 μm (35), which is similar to the capsule thickness observed in rats (**Figure 3**). Therefore, genetic background and capsule composition most likely play a major role in determining capsule thickness and overall whole lens mechanical stiffness. It is also plausible that the relative contributions of microstructure components to lens mechanical stiffness vary across species. For example, the thicker lens capsules observed in guinea pigs may contribute to increased lens stiffness (**Figure 3**), while reduced fiber cell compaction, as evidenced by wider fiber cells, may counteract the stiffness provided by thicker lens capsule. Thus, despite the thinner lens capsule in rats, the increased fiber cell compaction indicated by reduced fiber cell width may enhance resistance to lens deformation under compressive loads, thus resulting in lenses with similar stiffness in rats and guinea pigs.

The suture organization was similar between mice, rats, and guinea pigs (**Figure 5**). While the relationship between suture organization and lens biomechanics has not been examined in detail, previous research suggests that altered suture organization in EphA2 and EphA5 null mouse lenses may enhance lens shape recovery after coverslip removal (36). In our study, lenses from all three species nearly completely regained their original shape following coverslip removal, suggesting similar elastic properties (**Figure 2D**). Given that the overall suture morphology appears similar among mice, rats, and guinea pigs, and all three exhibit comparable resilience (**Figures 2D**, **5**), it is unlikely that suture organization significantly influences lens mechanical properties in these species.

Fiber cells in the innermost center (nuclear) regions of the lens become compacted (14, 37), forming a hardened spherical nuclear structure in mouse, bovine, sheep, and aged human lenses (27, 38–40). The lens nucleus has long been suggested as one of the main determinants of lens biomechanical properties. This idea is suggested by observations that increased lens nuclear stiffness and nuclear size parallel elevated whole lens stiffness with age in mice and humans (13, 14). Just as the lens biomechanics of guinea pigs and rat lenses are not different, the lens nucleus size is not different between rat and guinea pig lenses, while the size of the mouse lens nucleus is significantly smaller (**Figures 1C, F**, **2**). However, the nuclear fraction is not different between mouse and guinea pig lenses, despite the mouse lens being less stiff than the guinea pig lens. On the other hand, the rat lens nuclear fraction is significantly higher than that of the mouse lens, correlating with increased stiffness of the rat with respect to the mouse lens (**Figure 1G**). Thus, there is no consistent relationship between lens nuclear size and lens stiffness across these three species.

Overall, our findings indicate that lens stiffness arises from a complex interplay of multiple structural features rather than being determined by one factor alone, and lens morphometrics does not appear to provide a simple explanation of differences in lens biomechanical stiffness between these three species (**Figure 6**). Previous work has shown that loss of actin cytoskeleton components such as Tropomyosin (Tpm) 3.5, Ankyrin B, and Tropomodulin (Tmod) 1 reduce lens biomechanical stiffness by disrupting F-actin networks (27, 40–43). Additionally, knockout of specialized intermediate filament protein CP49 (phakinin) and deletion of both CP49 and Tmod1 reduces mouse lens stiffness (27, 44). These findings suggest that the cytoskeleton organization is a key determinant of lens mechanical properties and may differ among these three species.

Lens stiffness is also affected by hydrostatic pressure, maintained by sodium channels, gap junctions, and water channels and increased with aging (45–47). Previous studies have shown loss of connexin and aquaporin 0 disrupts the lens hydrostatic pressure and causes altered lens mechanical stiffness (46, 48–50). Interestingly, intracellular hydrostatic pressure gradients from different-sized lenses from 4 different species (mice, rats, rabbits, and dogs) show that the intracellular pressure gradient decreases as lens size increases (51). However, when the pressure was graphed as a function of normalized distance from the lens center to account for lens size differences, the intracellular pressure gradient was indistinguishable between all four species (51). This suggests that different-sized lenses possibly have different numbers of open ion channels that regulate hydrostatic pressure, which could affect lens stiffness in differently-sized lenses. Future studies should focus on comparing the actin cytoskeleton structure and organization in addition to hydrostatic pressure gradients between these different rodent species.

This is the first study to systematically compare lens morphometrics parameters and lens stiffness among differently sized and shaped lenses from three different species. Mice, rats, and guinea pigs all reach sexual maturity and are considered adults between 8 to 10 weeks (52–55). While our study accounted for biological age, the time to maturity and overall lifespan of each species may influence the development and structural properties of the lens. For instance, while mice and rats have relatively short lifespans of around 2-3 years and reach maturity rapidly, guinea pigs have longer lifespans and slower developmental trajectories. These differences in growth and aging patterns could contribute to variations in lens morphometry and stiffness (53, 54, 56, 57).

A limitation of our study is that the compressive coverslip assay was originally developed and optimized for mouse lenses (28). As the coverslips are added at a slightly oblique angle, the contact between the coverslip and the lens anterior surface may vary across different-sized lenses (28). It raises the possibility that these different-sized lenses with different radii of curvature are not experiencing similar loads. We have previously examined how mouse lens microstructure responds to different degrees of coverslip compression, where addition of 5-10 coverslips (23-29% axial strain) resulted in a significant increase in the epithelial cell area, widening of peripheral fiber cells, flattening of the capsule and increased suture gap, suggesting that the mechanical load was transferred to lens microstructures (26). It is worth noting that the degree of changes in these microstructural features in mouse lenses depended on the number of coverslips, in which higher loads cause increased changes (26, 58). In the future, a comparison of the lens microstructures under different degrees of coverslip compression in mice, rats, and guinea pigs may provide insight into the relative extents of load transfer for these lenses and the effectiveness of the coverslip method to study the stiffness of large lenses. In addition, alternative methods not dependent on lens size, such as laser-induced microbubble-based assay, atomic force microscopy, spinning lens test, stress-strain measurement systems, and mechanical lens stretching devices, could be used in the future to compare biomechanical properties (16, 20, 59–62).

In summary, we explored how lens morphometrics and microstructural characteristics relate to its mechanical properties. Our findings suggest that determinants of lens stiffness are complex and not determined by one single component. Future research is needed to further elucidate molecular components and other morphological attributions determining lens stiffness among different species.

## Data Availability

The original contributions presented in the study are included in the article/**Supplementary Material**. Further inquiries can be directed to the corresponding authors.
