# Supplementary material for: Comparative analysis of rodent lens morphometrics and biomechanical properties
Source: Front Ophthalmol (Lausanne). 2025 Apr 4;5:1562583. doi: 10.3389/fopht.2025.1562583 (PMC12006193; doi:10.3389/fopht.2025.1562583)
Supplement: Supplementary file 1 [file DataSheet1.docx]

Supplementary Material

# Supplementary Figures and Tables


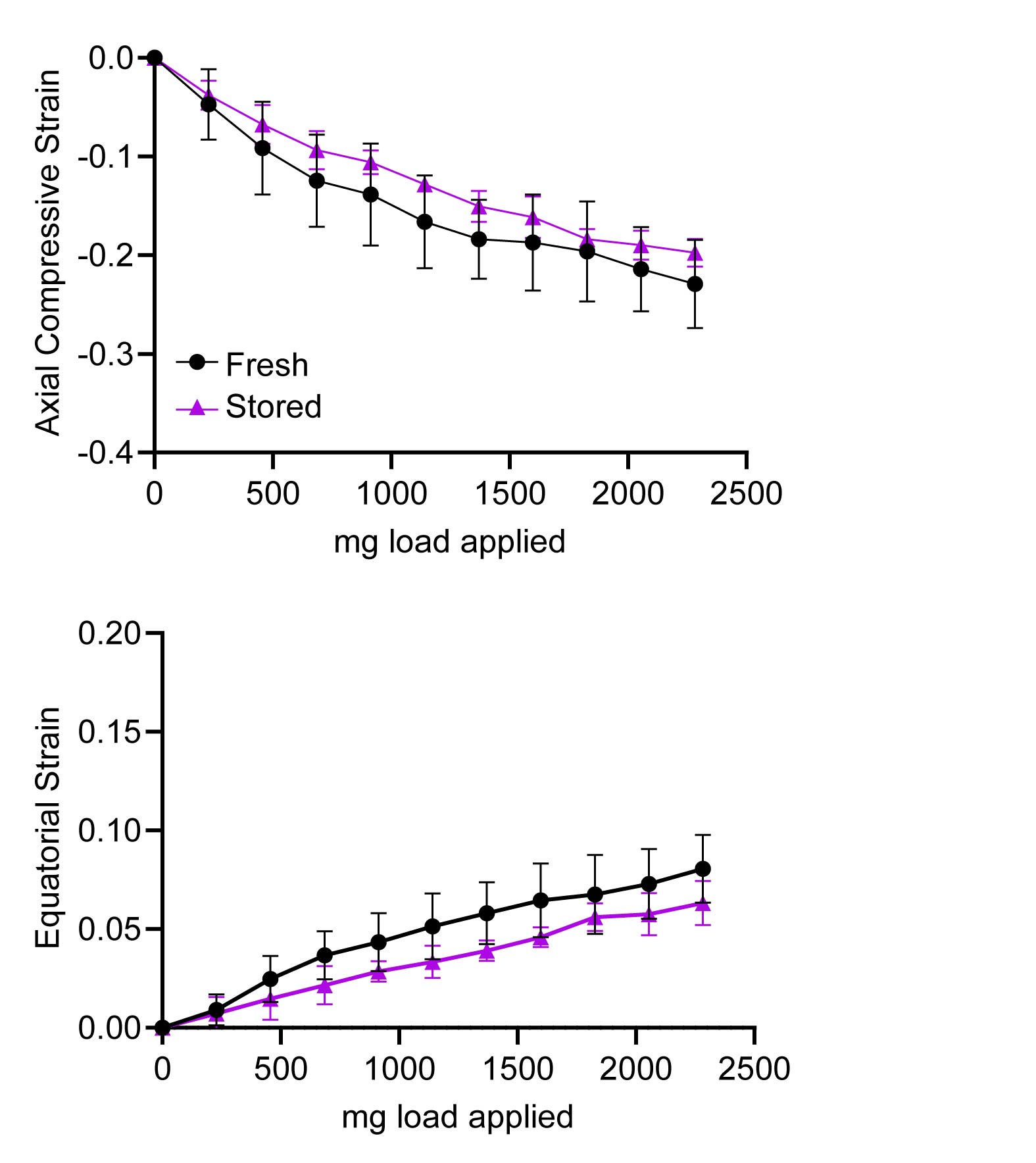


**Supplementary Figure 1:** **Freshly dissected vs stored lenses have similar biomechanical properties.** Sequential coverslip compression assay was performed on either freshly dissected mouse lenses or mouse lenses stored overnight in cold PBS. Both calculated axial and equatorial strain show no significant differences. N=10 (fresh) and 4 (stored).
